# TMS use in Depressive disorder in Youth

**DOI:** 10.1192/j.eurpsy.2022.1908

**Published:** 2022-09-01

**Authors:** A. Wadhwa, A. Sareen, Y. Saade

**Affiliations:** 1 University of Alabama at Birmingham, Child And Adolescent Psychiatry, Birmingham, United States of America; 2 Bronx Care Health System-Affiliated with the Icahn School of Medicine at Mount Sinai, Psychiatry, New York, United States of America; 3 Children’s National Hospital, Child And Adolescent Psychiatry, Washington DC, United States of America

**Keywords:** TMS, Depression, Child and adolescent psychiatry, neuromodulation

## Abstract

**Introduction:**

Trans-cranial magnetic stimulation (TMS) as a non-invasive method of altering brain activity (1) has widened the array of therapeutic options available for various psychiatric disorders.

**Objectives:**

Trans-cranial Magnetic stimulation (TMS) as a non-invasive method of altering brain activity has widened the array of therapeutic options available for various psychiatric disorders. •A large number of studies have shown therapeutic benefits in a wide range of patient population with majority of studies in adults. •TMS is used increasingly for the treatment of child and adolescent depression. •Yet, the scarcity of studies and lack of published guidelines for this population is notable. •As TMS use is expanding in this population, an overview of the use of TMS in children and adolescents with depression may provide much needed and timely perspective on this neuropsychiatric intervention.

**Methods:**

We searched all published studies using PubMed database, on TMS use in depressive disorders in children and adolescents. A total of 13 studies were found to have reported use of TMS in depression in children and adolescents.

**Results:**

We found various case series, open label studies as well as sham controlled blind studies indicating that TMS has been effective in treating depression in children and adolescents. No significant side effects were found in our review.

**Conclusions:**

Studies have shown that TMS is an effective treatment option for depressive disorders in children and adolescents. Initial studies look promising but implications in large pediatric population may be different and there is a need for more double blind, controlled trials with larger sample size.

**Disclosure:**

No significant relationships.

